# Genomic structure, phenotypic variation and drivers of diversification in *Euphonia hirundinacea* (Aves: Fringilidae)

**DOI:** 10.7717/peerj.20916

**Published:** 2026-03-24

**Authors:** Melisa Vázquez-López, Luz E. Zamudio-Beltrán, Sandra M. Ramírez-Barrera, Patricia Escalante-Pliego, Blanca E. Hernández-Baños

**Affiliations:** 1 Posgrado en Ciencias Biológicas, Universidad Nacional Autónoma de México, Mexico City, Mexico; 2 Museo de Zoología, Departamento de Biología Evolutiva, Facultad de Ciencias, Universidad Nacional Autónoma de México, Mexico City, Mexico; 3 Instituto de Ciencias de la Atmósfera y Cambio Climático, Universidad Nacional Autónoma de México, Mexico City, Mexico; 4 Instituto de Biología, Universidad Nacional Autónoma de México, Mexico City, Mexico

**Keywords:** *Euphonia hirundinacea*, Phylogeography, Phenotypic variation, Mesoamerica, Isolation by distance, Sex-biased traits, Climatic fluctuations

## Abstract

The evolutionary drivers of genetic and phenotypic diversity in widespread Neotropical birds remain poorly understood, particularly in species with high dispersal capabilities. In this study, we examine the population genetic structure, demographic history, and phenotypic variation of *Euphonia hirundinacea* across its Mesoamerican range using NextRad genomic data, as well as geographic and phenotypic variables. A maximum-likelihood phylogeny confirmed the monophyly of *E. hirundinacea*, with two well-supported clades corresponding to geographically segregated populations that align with the subspecies *E. h. hirundinacea* and *E. h. gnatho*. Our findings indicate moderate genetic differentiation partially shaped by isolation by distance (IBD), with the Nicaragua Depression acting as a semi-permeable barrier that allows limited gene flow. Most phenotypic traits did not correlate with genetic divergence, geographic distance, or environmental variables. However, in females, wing chord and throat coloration were significantly associated with both genetic and geographic distances. Demographic analyses support a scenario of divergence initiated during Pleistocene climatic fluctuations (∼2,668,739.13 years ago), followed by population expansion during Holocene (∼20,000 years ago) and secondary contact, supported by ecological niche models. The species’ ecological flexibility and high vagility likely mitigated long-term isolation and facilitated rapid post-glacial recolonization. Overall, our findings highlight the roles of isolation by distance, Pleistocene climatic dynamics, geographic barriers, and sex-specific traits in shaping diversification in a highly mobile Neotropical bird.

## Introduction

Phylogeographic studies have documented the evolutionary processes and biogeographic patterns shaping the genetic structure of Neotropical bird populations (Ramírez-Barrera et al., 2018; Sánchez-González et al., 2021; Smith et al., 2014; Weir, 2006). These studies have highlighted the influence of both ecological (*e.g.*, Lavinia et al., 2019) and geographical barriers (Barber & Klicka, 2010; *e.g.*, Barrera-Guzmán et al., 2012), the role of Pleistocene climatic changes, in promoting genetic differentiation by restricting gene flow (*e.g.*, Rodríguez-Gómez & Ornelas, 2018), as well as the species-specific dispersal capabilities in the context of landscape features (Batalha-Filho et al., 2024).

In Mesoamerica, phylogeographic studies have revealed that historical climatic dynamics and complex topography have played key roles in shaping avian genetic structure (Espinosa-Chávez et al., 2024; Zamudio-Beltrán et al., 2020a). The Isthmus of Tehuantepec, for instance, has acted as a major biogeographic barrier for highland species (*e.g.*, Zamudio-Beltrán et al., 2020a; Zamudio-Beltrán et al., 2020b) and for some lowland birds (*e.g.*, Vázquez-López et al., 2021). Comparably, the Nicaraguan Depression has acted as an ecological and genetic barrier for lowland species, both during the Last Glacial Maximum (LGM) and under current environmental conditions (*e.g.*, Castillo-Chora et al., 2021). During the LGM, shifts in vegetation and ecological conditions likely altered habitat suitability, driving scenarios of population fragmentation and/or population expansions in various species (Zamudio-Beltrán et al., 2020a; Miller et al., 2011; Rodríguez-Gómez et al., 2021; Vásquez-Aguilar et al., 2023).

*Euphonia hirundinacea* is a widely distributed species, ranging from the south of Tamaulipas (Mexico) to western Panama (0–1,800 m). According to previous phylogenetic studies, *Euphonia hirundinacea* is closely related to other members of Euphoniinae subfamily: *E. laniirostris*, *E. violacea*, and *E. chalybea* which diverged from *E. minuta* around ∼2 million years ago (Imfeld, Barker & Brumfield, 2020; Vázquez-López et al., 2024). Although the phylogenetic relationships among Euphoniinae species have been resolved (Imfeld, Barker & Brumfield, 2020; Vázquez-López et al., 2024), there is only limited knowledge of intraspecific variation, population structure, and species limits within *Euphonia*. Recent studies suggest that additional cryptic lineages may exist, warranting further investigation (Imfeld, Barker & Brumfield, 2020; Vázquez-López et al., 2020; Vázquez-López et al., 2024). The biogeographic framework of birds inhabiting Mesoamerica, the biological characteristics of this species, and the lack of knowledge of the evolution at intraspecific level make *Euphonia hirundinacea* an ideal model to explore how historical and ecological factors may be influencing population differentiation structure patterns in the Mesoamerican lowlands.

The Yellow-thorated Euphonia, *E. hirundinacea* exhibits remarkable ecological flexibility, inhabiting a variety of Mesoamerican lowland habitats, including tropical deciduous and rain forests, gallery forests, secondary growth forests, gardens, and even tree patches in pastures and plantations (Howell & Webb, 1995; Whitman, 2020). *Euphonia hirundinacea* displays phenotypic variation across its range, with two recognized subspecies: *E. h. hirundinacea*, distributed from Mexico to southeastern Nicaragua, and *E. h. gnatho*, distributed from northwestern Nicaragua to western Panama. According to Storer (1970) the subspecies *E*. *h*. *gnatho* hybridizes with the nominal subspecies in the southeastern of Guatemala. Males of *E*. *hirundinacea* have the typical coloration pattern of yellow-throated euphonias, with bluish-black coloration on the back and yellow underside, including the throat. The females are olive green, with the forecrown and upper anterior areas yellowish olive green (Whitman, 2020). Phenotypic variation in *E. hirundinacea* is not discrete; instead, it appears as a continuum, with gradual changes in coloration across its geographic range. However, there are notable phenotypic differences in birds south of the Nicaragua Depression to Costa Rica, which corresponds to the distribution described for *E. h. gnatho*. Males of *E. h. gnatho* are larger than *E. h. hirundinacea* males, with a more greenish back and less bluish gloss on the upperparts (Hellmayr, 1936), while females have entirely yellow throat and breast and yellower upper parts (Phillips, 1966). Females from Chiapas to the Pacific Slope also have a yellow throat and breast. Although other subspecies (*E. h. ruselli, E. h. caribbea,* and *E. h. suttoni*) have been proposed, they have not been formally recognized by taxonomic authorities.

This study aims to explore the genetic and morphological variation in *Euphonia hirundinacea* and its association with geographic barriers, ecological conditions, and geographic distance. Specifically, we aimed to: (1) evaluate the genetic structure of *Euphonia hirundinacea* across its distribution; (2) determine whether the Nicaragua Depression acts as a genetic barrier; (3) explore the role of environmental suitability in demographic changes and population structure using geographic projections in current and past scenarios; (4) explore the role of geographic distance in genetic differentiation; and (5) determine the role of geographic distances and climatological factors in phenotypic variation. By integrating genetic and phenotypic data, we aim to propose a hypothesis of evolutionary and ecological drivers of variation in *Euphonia hirundinacea* and contribute to a broader understanding of the diversification of Mesoamerica lowland birds.

## Material and Methods

### Tissues sampling

**Figure 1:**
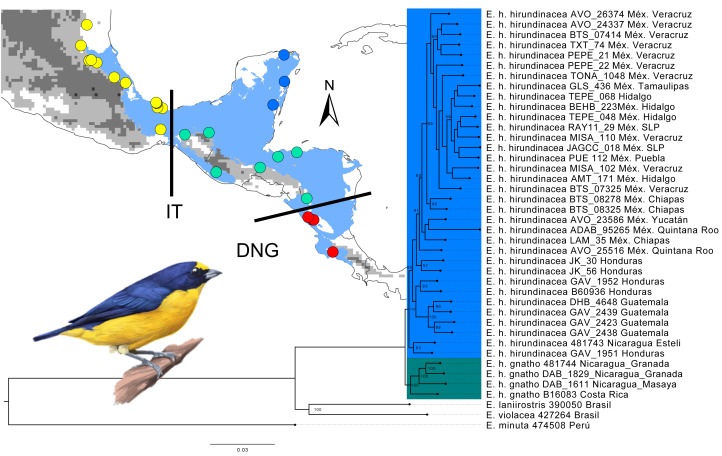
Maximum-likelihood tree using SNP data set in RAXML, geographic distribution, and sampling tissues of *Euphonia hirundinacea*. The green clade represents the sampling assigned to *Euphonia h. gnatho*, and the blue clade represents the sampling assigned to *Euphonia h. hirundinacea*. Node support values (only those >80) are shown. Light blue indicates the potential distribution of *Euphonia hirundinacea* according to Navarro-Sigüenza et al. (2018). The gray scale represents the elevation gradient (NASA Shuttle Radar Topography Mission (SRTM), 2013). The points are the geographic sampling locations for tissues. The color of the points shows the preassigned populations: Yellow (NI: North of Isthmus of Tehuantepec), Blue (YUC: Yucatan Peninsula), Green (SI: South of Isthmus of Tehuantepec), and Red (DNG: Nicaragua Depression to Costa Rica). *Euphonia hirundinacea* Illustration by Germán García Lugo.

We obtained 38 samples of *Euphonia hirundinacea* from Museum of Natural Science (Louisiana State University) (LSUMN), Field Museum of Natural History (FMNH), UWBM, and Museo de Zoología de la Facultad de Ciencias (MZFC) (See Fig. 1 and Table 1, collection permit: Instituto Nacional de Ecología, SEMARNAT, México FAUT-0169). The samples were taken from different regions: Eastern/Northern Isthmus of Tehuantepec (Tamaulipas, Veracruz, San Luis Potosí, Puebla, Hidalgo), Southern Isthmus of Tehuantepec (Chiapas to Nicaragua), Yucatan Peninsula, and the Nicaragua Depression to Costa Rica. We also took one tissue sample for two species to act as sister groups and one outgroup species: *Euphonia violacea* (Accession number SAMN30618678), *Euphonia laniirostris* (Accession number: SAMN30618674), and *Euphonia minuta* (Accession number: SAMN30630219) (outgroup).

**Table 1:** Tissues sampling for genetic analysis of *Euphonia hirundinacea*, sister groups (*Euphonia violacea*, *Euphonia laniirostris*) and outgroup (*Euphonia minuta*).

Latitude	Longitude	Species	Catalogue number	Locality	Collection
16.77870	−93.4193	*E. h.hirundinacea*	BTS_08278, BTS_08325	Mexico, Chiapas, Ocozocuautla, 5 km S.	UWBM
16.88740	−92.0206	*E. h.hirundinacea*	LAM_35	Mexico, Chiapas, Camino a Zona Arqueológica de Toniná, Rancho los Abuelos.	MZFC
15.75971	−86.917	*E. h.hirundinacea*	GAV_1951, GAV_1952	Honduras, Departamento de Atlántida , La Ceiba, 15 km W.	UWBM
14.86700	−89.05	*E. h.hirundinacea*	JK_30, JK_56	Honduras, Departamento de Copán, Copán Ruinas, 15 km ENE.	UWBM
15.50278	−88.01361	*E. h.hirundinacea*	B_60936	Honduras, Departamento de Cortés, .	LSU
13.08333	−86.35	*E. h.hirundinacea*	481743	Nicaragua, Departamento de Esteli, Reserva Natural Miraflor-Morpotente, Finca Neblina del Bosque, 20 km NNE Esteli.	FMNH
14.60000	−91.612	*E. h.hirundinacea*	DHB_4648, GAV_2423, GAV_2438, GAV_2439	Guatemala, Departamento de Retalhuleu, San Felipe Retalhuleu, 5 km S; Finca El Nino.	UWBM
21.06667	−98.98733	*E. h.hirundinacea*	AMT_171	Mexico, Hidalgo, El Coyol, campamento.	MZFC
20.95000	−98.55	*E. h.hirundinacea*	BEHB_226	Mexico, Hidalgo, Pilcuatla.	MZFC
21.09333	−98.85333	*E. h.hirundinacea*	TEPE_048, TEPE_068	Mexico, Hidalgo, Texcapa.	MZFC
17.07500	−94.84	*E. h.hirundinacea*	TONA_1048	Mexico, Oaxaca, 16 km ENE Piedra Blanca.	MZFC
20.09167	−97.51667	*E. h.hirundinacea*	PUE_112	Mexico, Puebla, Cuitchat, 8 km NE Cuetzalan.	MZFC
18.50361	−88.30538	*E. h.hirundinacea*	ADAB_95265	Mexico, Quintana Roo, Chetumal.	CNAV
19.85095	−87.63934	*E. h.hirundinacea*	AVO_25516	Mexico, Quintana Roo, Reserva Biosfera Sian Kaan, Rancho Viejo.	CNAV
21.93610	−99.4616	*E. h.hirundinacea*	RAY11_29, JGCC_018	Mexico, San Luis Potosí, Las Guapas, Los Cuartos.	MZFC
14.60000	−91.6116	*E. h.hirundinacea*	GLS_436	Mexico, Tamaulipas, El Mante, El Encino.	MZFC
18.58333	−95.05	*E. h.hirundinacea*	AVO_24337, AVO_26374	Mexico, Veracruz, Los Tuxtlas, 200 m Estación de Biología Tropical.	CNAV
18.63995	−95.09344	*E. h.hirundinacea*	BTS_07325	Mexico, Veracruz, Montepío.	UWBM
18.62188	−95.0729	*E. h.hirundinacea*	BTS_07414	Mexico, Veracruz, Balzapote, Finca La Iguana.	UWBM
19.76994	−96.86208	*E. h.hirundinacea*	MISA_102, MISA_110	Mexico, Veracruz, Villa Nueva, 800 m al SE.	MZFC
18.35611	−95.01861	*E. h.hirundinacea*	PEPE_21, PEPE_22	Mexico, Veracruz, Comunidad Benito Juárez 1.2 km SE.	MZFC
18.30542	−94.72444	*E. h.hirundinacea*	TXT_074	Mexico, Veracruz, San Martín Pajapan.	MZFC
21.40083	−87.67777	*E. h.hirundinacea*	AVO_23586	Mexico, Yucatán, Reserva de Ría Lagartos, 12 Km S El Cuyo.	CNAV
11.83400	−85.995	*E. h. gnatho*	DAB_1829	Nicaragua, Departamento de Granada, Mombacho Volcano 48km SE, Managua.	UWBM
11.83758	−85.95089	*E. h. gnatho*	481744	Nicaragua, Departamento de Granada, Reserva Natural Volcan Mombacho, 11km S Granada.	FMNH
11.99800	−86.278	*E. h. gnatho*	DAB_1611	Nicaragua, Departamento de Masaya, Las Nubes.	UWBM
9.97625	−84.83836	*E. h. gnatho*	B16083	Costa Rica, Provincia Puntarenas, 3 km E Punta Leona.	LSU
		*E. laniirostris*	390050	Brasil	LSU
		*E. violacea*	427264	Brasil	FMNH
		*E. minuta*	474508	Perú	FMNH

### Laboratory procedures and sequence preparation for nextRAD sequencing

We extracted genomic DNA using the DNeasy tissues kit (Qiagen, Hilden, Germany) and phenol:chloroform tissue protocol. DNA quality was verified using gel electrophoresis and the concentration was determined with a Qubit 3 fluorometer. The RAD sequence data were obtained from the company SNPsaurus (http://snpsaurus.com/) using the nextRAD technique (Russello et al., 2015). The nextRAD libraries were sequenced on a HiSeq 4000 with one lane of 150 bp reads (University of Oregon). The raw data obtained are available on GenBank in the project: PRJNA1292171.

### Quality filtering and *de novo* alignment

We used iPyrad (Eaton & Overcast, 2020) to filter and align the raw reads, retaining reads with a minimum length of 100 base pairs and a Phred Q score of 43. We optimized the cluster threshold value to avoid paralogues (McCartney-Melstad, Gidiş & Shaffer, 2019), using the 92% cluster threshold. Only loci with two alleles were retained. The maximum fraction of heterozygous bases allowed in consensus seqs was 0.05, default value of iPyrad. Then, we retained only loci that were present in at least 50% of individuals and excluded samples with excessive (>50%) missing data. The maximum number of SNPs allowed in the final loci were indicated at 0.2 following the recommendations of iPyrad. Finally, with VCFtools (Danecek et al., 2011) we filtered the SNPs with a minor allele frequency (MAF) of 0.05.

### Phylogenetic analysis and genetic population structure

We performed a phylogenetic analysis based on Maximum Likelihood (ML) with the SNPs alignment using RaxML v.8.0.0 (Stamatakis, 2014) in the CIPRES Science Gateway (Miller, Pfeiffer & Schwartz, 2012), using the GTRGAMMA nucleotide substitution model with RAxML and automatic bootstrapping. To characterize patterns of genetic structure across the sampling range, we tested four possible populations that correspond with the principal distribution breaks populations previously reported for Mesoamerica lowlands: (1) North of the Isthmus of Tehuantepec (NI), including Tamaulipas, San Luis Potosí, Hidalgo, Puebla and Veracruz; (2) Yucatan Peninsula (YUC); (3) South of the Isthmus of Tehuantepec (SI) from Chiapas to northern Nicaragua; and (4) Nicaragua Depression to Costa Rica (DNG). DNG also corresponds with the distribution of the recognized subspecies *E. h. gnatho*. To identified and visualized population structure we conducted a principal components analysis (PCA) using the R package SNPRelate (Zheng et al., 2012). We also tested the genetic structure using STRUCTURE 2.3 (Pritchard, Stephens & Donnelly, 2000) using the admixture model with correlated allele frequencies and prior information regarding sampling locations. We used StrAuto independent runs to test the genetic clusters (*K* = 1 –5) with five independent runs of 50,000 MCMC cycles after a burn-in period of 2,000 generations. We evaluated the best K using the Evanno, Regnaut & Goudet (2005) method, which finds the number of clusters that maximizes the second-order rate of change of the log probability of the data (ΔK).

### Current and past distribution and population demographic changes

We performed Ecological Niche Models to infer the suitability of environmental conditions under present scenarios and past scenarios. Presence records of *Euphonia hirundinacea* were obtained from the Global Biodiversity Information Facility (http://www.gbif.org/) (Table S1). We cleaned the data following the steps of Simões et al. (2020). This resulted in a total of 413 records (Table S1). We used current bioclimatic layers (Karger et al., 2017) and past climatic layers from the Paleoclim database (Hijmans et al., 2005). Past scenarios were projected onto three different periods: the Last Interglacial (130 kya) (Otto-Bliesner et al., 2006), the Last Glacial Maximum (21 kya) (Karger et al., 2017), and the Early, Medium and Late Holocene (6 kya) (Fordham et al., 2017). We selected the most important and non-redundant (Pearson correlation coefficient <0.75) BIO variables from the first three principal components of a PCA of the environmental variables. Finally, we performed the ENM analysis with the next five variables: BIO 3 (Isothermality), BIO 4 (Temperature seasonality), BIO 6 (Min Temperature of Coldest Month), BIO 12 (median annual precipitation) and BIO 17 (Precipitation of Driest Quarter). The “M” area was defined with a convex hull of the occurrence points and a 25 km buffer, using the script from Simões et al. (2020) and the ellipsenm R package (Cobos et al., 2020). To ensure that the M area was confined to the ecoregions where *E*. *hirundinacea* has its known distribution, we visually verified it by overlaying the final M polygon with the WWF ecoregion map in R. The Ecological Niche Modeling and past projections were performed in Maxent v3.4.1 with 10 cross validation replicates and extrapolation.

For demographic characterization, we constructed Extended Bayesian Skyline Plots (Drummond et al., 2005) in BEAST 2.7.7 (Bouckaert et al., 2019) to infer changes in demography over time, using the SNP matrix, converted to nexus format. We set the HKY model evolution, a strict molecular clock and Extended Coalescent Bayesian Skyline as the tree prior. The analyses were run for 20,000,000 generations, sampling every 1,000 generations, with a burn-in of 20 percent, finally in Tracer version 1.6 (Rambaut et al., 2018) we reviewed that the value of effective sample size (ESS) was > 200.

We used the Generalized Phylogenetic Coalescent Sampler (G-PhoCS; Gronau et al., 2011), a full-likelihood method based on the multispecies coalescent model, to estimate key demographic parameters, including mutation-scaled ancestral population size (*θ*), migration rate (m), and divergence time (*τ*). For Tau and Theta, we tested the priors alpha = 4 and beta = 800, and for migration rate we tested migration rate alpha = 0.5 and migration rate beta = 10. For the ancestral population we used the prior tau-beta=20,000. We performed 1,000,000 MCMC iterations, sampling every 1,000 generations, we reviewed that the ESS values were > 200 using Tracer version 1.6 (Rambaut et al., 2018) with a burning of 20 percent. To convert the Tau (*τ*) values into years, we used the formula T = *τ*/µ, where µ is the mutation rate per year. This rate was derived from the germline mutation rate for the collared flycatcher (*Ficedula albicollis*), estimated at 4.6  × 10^−^^9^ mutations per site per generation (Smeds, Qvarnström & Ellegren, 2016). Given that the study associated this rate with a 2-year generation time, and assuming the same generation time for our study species (IUCN, 2020; BirdLife International, 2020; Bird et al., 2020), we calculated a mutation rate per year of *μ* = 2.3 ×10^−^^9^. The theta values (*θ*) were converted to effective population size using *θ* = 4Neµg, where Ne is the effective population size, µ is mutations per nucleotide site per generation and g the average generation time in years. Finally, we obtained the proportion of migration between the both populations with msx (Msx = msx × *θ*x/4). Also, we tested the potential gene flow between the two groups with the ABBA-BABA test (Green et al., 2010; Durand et al., 2011) using the scripts available at https://github.com/millanek/Dsuite. We fixed P1 the sister group as *Euphonia laniirostris*, P2 as *E. hirundinacea gnatho* and P3 as *Euphonia h. hirundinacea*. With this arrangement we calculated the Patterson’s D statistic. The test counts the number of ABBA patterns (where P2 and P3 share a derived allele) and BABA patterns (where P1 and P3 share a derived allele). Following the author’s script, we then calculated the admixture proportion and dived the *E. h. hirundinacea* samples to set P3a and P3b.

### Morphometric analysis

We collected data on six morphometric traits of 75 (37 females and 38 males) study skins from two museums: the American Museum of Natural History (AMNH), and Colección Nacional de Aves IB-UNAM (CNAV) (Table S2). The measured specimens were distinct from the genetic data set because skins were not accessible for most of our genetic samples; however, only a few individuals from the CNAV collection representing *E*. *hirundinacea hirundinacea* possess combined genetic, morphometric, and coloration data. To avoid measurement bias, all data were collected by the first author across three independent trials. To measure wing chord (WC) and tail length (TLE), we used an Avinet calibrated ruler to the nearest 0.5 mm. Additionally, we measured culmen length (CL), tarsus length (TL), and beak depth (BD) with a Mitutoyo digital caliper to the nearest 0.1 mm. We checked the normality of the data by performing the Shapiro test; since the data followed a normal distribution, we analyzed the variance using the Bartlett test. Then, we tested sexual dimorphism in the six morphometric characters with a Student’s *T*-test. Since our data revealed sexual dimorphism in five morphometric variables: (Table S3), the posterior statical analysis were done with separated sex. We defined the groups to compare based on the results of the phylogenetic analysis and population structure analysis: *E. hirundinacea hirundinacea* and *E. h. gnatho* (see Results: 3.2 Phylogenetic inference and genetic structure). We compared these two groups using Student’s *T*-test and a multivariable analysis MANOVA. Finally, to identify the dispersion of points in a multivariable space we made a PERMANOVA and PCA. We performed all statistical analyses in R (R Core Team, 2020).

### Color data

We obtained plumage reflectance spectra for the following plumage patches: forehead, crown, upper back, throat, breast, belly, and undertail coverts, of 89 study skins (48 females and 41 males) (Table S2) held at the AMNH Ornithology Collection and Museo de Zoología de la Facultad de Ciencias (MZFC) and Colección Nacional de Aves (IB, UNAM). Color data were collected as previously described in Ramírez-Barrera et al. (2019), specifically, the plumage color was determined across all specimens using the Ocean Optics USB2000 spectrophotometer, which was coupled to an Ocean Optics PX-2 pulsed xenon light source and a split fiber-optic probe. The probe incorporated a rubber stopper; this addition served to exclude ambient light and maintain a constant distance and perpendicular (90°) angle between the probe tip and the feathers. Standardized protocols were applied (Eaton, 2005) when recording the reflectance capacity of the plumage at each wavelength, covering the avian visual band (300–700 nm). Finally, the spectrophotometric data were compiled and processed utilizing the software provided by Ocean Optics. Then, with the R package PAVO (Maia et al., 2019) we cleaned and processed the data to smooth the spectrographic curves and correct for electric noise. From the smoothed and noise-corrected spectrographic data of each feather patch we obtained the standard variables of hue (H3), chroma (S9) and brightness (B2). Then, we performed a PCA in the R package FactoMineR (Lê, Josse & Husson, 2008). Since this species presents strong sexual dimorphism, we processed and analyzed the color data separately by sex.

### Correlations among genetic, phenotypic, geographic, and environmental distances within *Euphonia hirundinacea*

We applied the MRM (multiple regression on distance matrices) analysis (Lichstein, 2007) with 1,000 permutations in R with the package Ecodist (Goslee & Urban, 2007) to evaluate the correlation of genetic variation with phenotypic variation, geographic distance and environmental distance within *E. hirundinacea*. First, we assigned the genetic distance matrix as the response matrix and the geographic distance matrix as the explanatory matrix. Then, to evaluate the phenotype and environment and its correlation with the genetic variation, we performed an MRM analysis using the wing chord (WC) and the PC1 of the color measurements, and Temperature and Precipitation as the explanatory variables. The genetic distances were calculated with the VCF file using the R package fastreeR (Gkanogiannis, 2024), using the function VCF2DIST which computes a cosine distance matrix. To obtain the matrix of geographic distances, we obtained the coordinates of every tissue sample and calculated the Euclidian distance using base R (R Core Team, 2020). Finally, we analyzed the importance of geography and environment for the phenotype variation using all of the individuals’ color and wing chord data. We constructed models with the variable’s brightness, hue and saturation for the back, throat, belly, and breast patches as response variables and geographic and environmental distances as the explanatory matrices. We calculated the distance matrices for wing chord, color variables, bioclimatic variables and geographic distances (Euclidian distance) with base R (R Core Team, 2020).

## Results

### Final data set

We obtained a total of 132,149 SNPs for 41 samples (including the outgroup) throughout the range of *Euphonia hirundinacea,* with this matrix we constructed the phylogenetic analysis. After applying a MAF of 0.05, we retained a database of 27,605 SNPs for 41 samples, which was used in the population genetic structure analyses.

### Phylogenetic inference and genetic structure

A maximum-likelihood tree was obtained using of 132,149 SNPs with high support for the branches (Fig. 1). The tree shows a monophyletic group of the members of the species (*Euphonia hirundinacea*) and two main groups within it. The genetic groups are geographically separated and corresponded to *E. h. hirundinacea* and *E. h. gnatho*. The *hirundinacea* and *gnatho* group had high support values (Bootstrap: 90 and 100 respectively). The maximum likelihood tree generated from the concatenated SNPs displayed a topology that was consistent with the PCA (see Fig. 1). There are two main clades observed in the tree, the first main clade is composed of samples collected from locations along the coast of the Gulf of Mexico (North of the Isthmus of Tehuantepec), the Yucatan Peninsula, and the group South of the Isthmus (Bootstrap: 80). This first clade aligns with the subspecies *E. h. hirundinacea*. The second main clade consists of samples from the Nicaragua Depression including one sample from Costa Rica (Bootstrap: 100). In accordance with the subspecies distribution, the samples from the second main clade can be grouped as *E. h. gnatho*. We also found substructure within the *E. h. hirundinacea* group, with two well-supported clades: one corresponded to the samples from North of the Isthmus of Tehuantepec (Bootstrap: 90), and the other corresponded to the samples from Guatemala (Bootstrap: 100), while the remaining samples did not exhibit any population substructure. These results support the consideration of *E. hirundinacea* as two independent evolutionary units.

**Figure 2:**
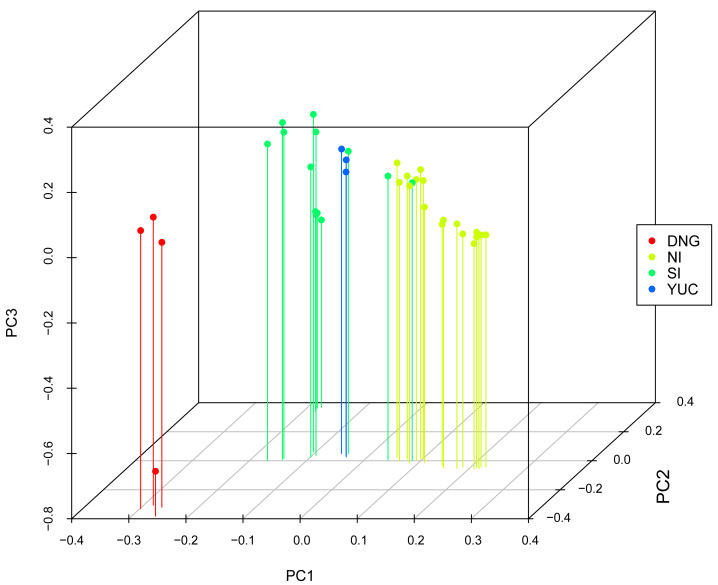
PCA of SNPs from pre-assigned populations of Euphonia hirundinacea. The axes represent scores in first three principal components (PC1: 8.1%, PC2: 5.1%, and PC3: 3.9% of variance). NI, North of Isthmus of Tehuantepec; YUC, Yucatan Peninsula; SI, South of Isthmus of Tehuantepec; DNG, Nicaragua Depression to Costa Rica.

The analysis of the genomic variation revealed that the first three principal components explained 17.1% of the total variation. Specifically, PC1 accounted for 8.1% of the variation, PC2 explained 5.1%, and PC3 explained 3.9%. The results show that the four Nicaragua Depression samples were distinct from the rest of the samples from northern populations (North of the Isthmus, South of the Isthmus, and Yucatan Peninsula; Fig. 2). The first and second principal components separated the four Nicaragua Depression samples from the rest, while the third principal component did not show any difference among populations.

**Figure 3:**
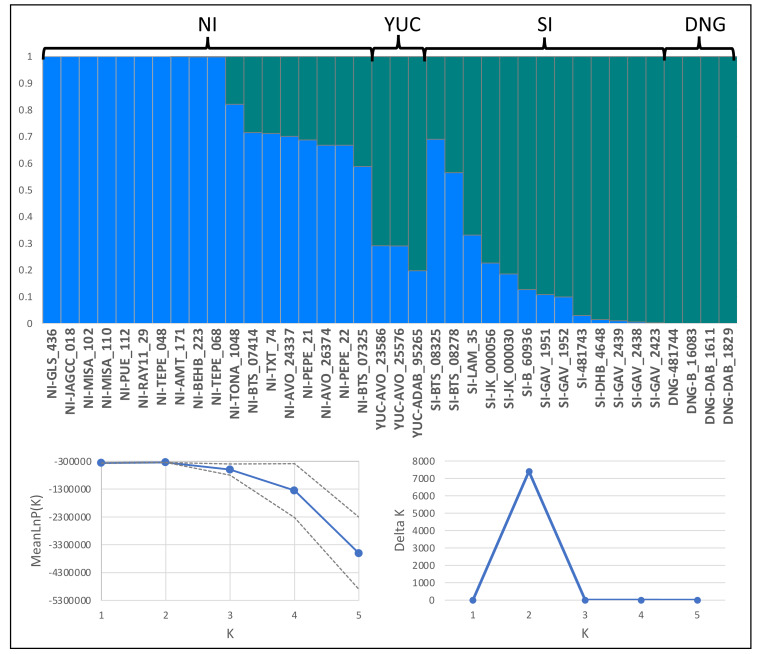
Structure analysis for *K* = 2. Bars represent estimated ancestry proportions for each genetic cluster, ordered by the four pre-defined populations: NI, North of Isthmus of Tehuantepec; YUC, Yucatan Peninsula; SI, South of Isthmus of Tehuantepec; DNG, Nicaragua Depression to Costa Rica; Left graphic, MeanLnP(K); Right graphic, Delta K.

Structure analysis supported two clusters (*K* = 2; Fig. 3) since log-likelihood and Delta K value are bigger for *K* = 2. The Structure graphic showed admixture between both populations for the samples of Yucatán population and some samples from North of Isthmus and South of Isthmus.

### Morphometric variation

Morphometric sexual dimorphism was identified in five variables (Table S3). Males differed significantly between *E. h. hirundinacea* and *E. h. gnatho* in tarsus length (TL) and wing chord (WC), with *E. h. gnatho* exhibiting larger median than *E. h. hirundinacea* (*p* < 0.05; Table 2). In contrast, females showed significant differences in tarsus length (TL) and bill depth (BD), also with *E. h. gnatho* having larger median than *E. h. hirundinacea* (*p* < 0.05; Table 2). The MANOVA test was significant for females (*p* < 0.05). Instead, for males the MANOVA test was not significant (*p* > 0.05) (Table S4).

**Table 2:** Differences between two groups: *E. h. hirundinacea* and *E. h. gnatho*.

Females				Males		
	*hirundinacea* (27 skins)	*gnatho* (10 skins)	*p-value*	*hirundinacea* (30 skins*)*	*gnatho* (8 skins)	*p-value*
TL	13.10	13.96	1.00E−03*	13.62	14.04	0.04*
CL	8.91	8.96	0.77	9.18	8.91	0.32
WC	58.38	59.53	0.14	59.51	61.57	0.02*
TLE	30.70	31.24	0.28	32.16	32.38	0.76
BD	5.15	5.45	3.69E−05*	5.32	5.38	0.69
BW	6.66	6.77	0.11	6.82	6.81	0.91

**Notes.**

TL Tarsus length CL Culmen Length WC Wing Chord TL Tail Length BD Bill Depth

The asterisks indicate statistically significant results with a *p*-value < 0.05.

**Figure 4:**
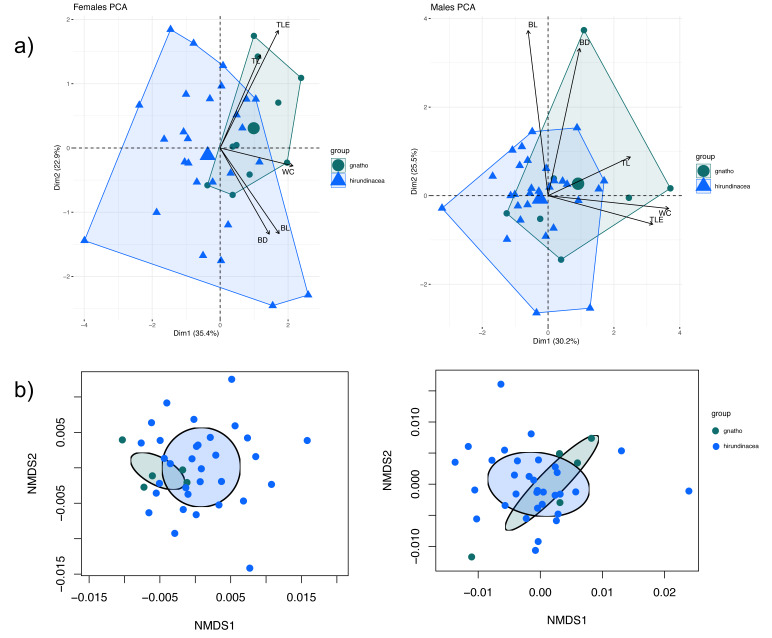
Principal Components Analysis and PERMANOVA of morphometric data. (A) PCA graphic, left females, right males. (B) NMDS graphic, left females, right males. Wing Chord (WC), Tail Length (TLE), Culmen Length (CL), Tarsus Length (TL), and Beak depth (BD).

The PERMANOVA test was not significant for females (*p* > 0.05), with a R^2^ of 0.05 for the model. For males the PERMANOVA test was significant (*p* < 0.05) with a R^2^ of 0.1 for the model (Table S4). PCA analyses for males showed that 74.65% of the variance was explained by the first three principal components, while for females, 78.23% of the variance was explained by the first three principal components. The most important variables in the first principal component were the wing chord and the tail length for males, and the wing chord and bill width for females (Table S5). Both, the dispersion plot of PCA and the NMDS plot for PERMANOVA showed overlap between *E. h. hirundinacea* and *E. h. gnatho* (Figs. 4A and 4B).

### Demographic results, population divegence time, current and paleo distribution

**Figure 5:**
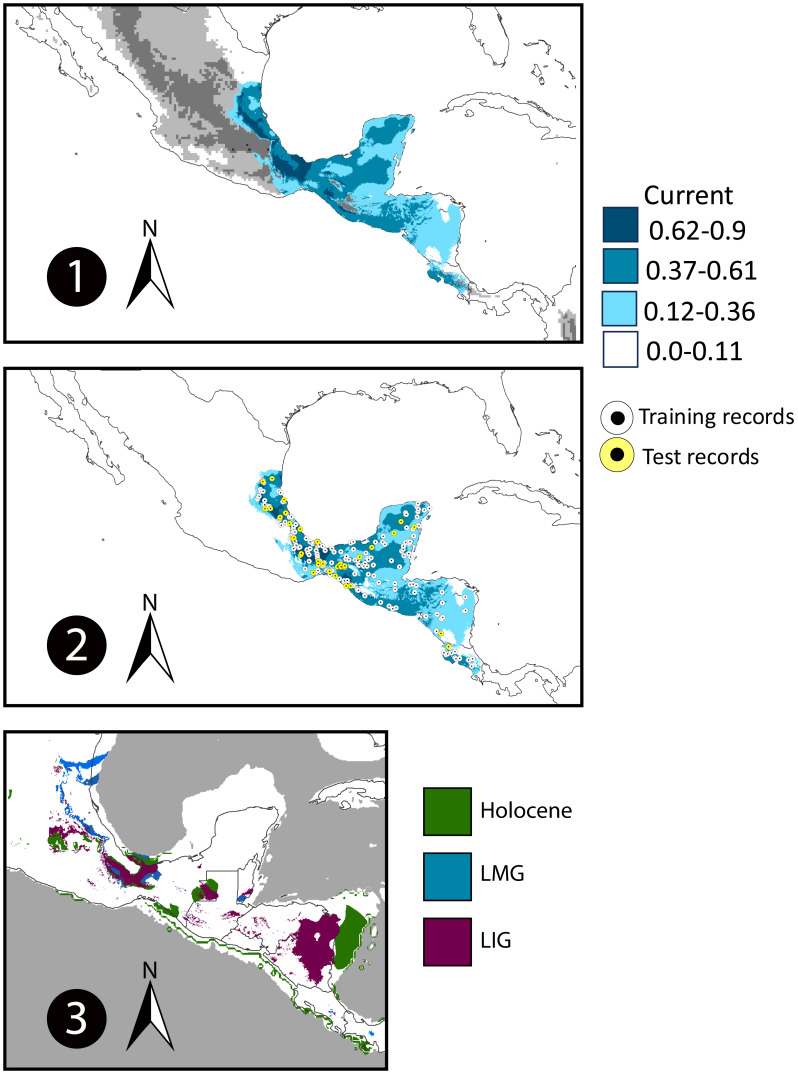
Current and paleo distributions. 1. Maxent environmental niche model projected onto the geography for *Euphonia hirundinacea*; the gray scale represents the elevation gradient (NASA Shuttle Radar Topography Mission (SRTM), 2013). 2. Maxent environmental niche model with the training and test records used to calibrate and evaluate the model. 3. Maxent environmental niche model projected for paleodistributions into the Global Climate Model for Holocene, Last Glacial Maximum (LGM), and Last Interglacial (LIG).

**Figure 6:**
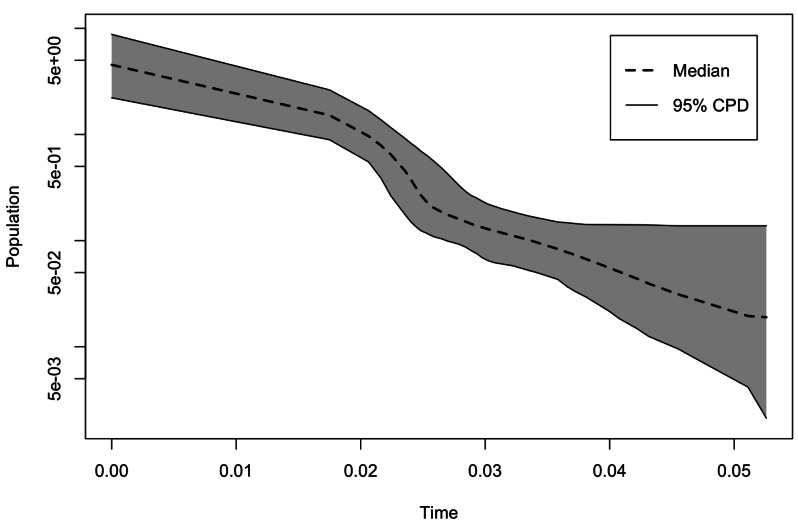
Extended Bayesian skyline plots. Time is indicated in millions of years ago (mya). Solid lines represent median estimates, with 95% confidence intervals shaded.

Our models obtained a high mean value for Area Under the Curve (AUC: 0.790), this indicates a good fit of the ENMs. The ENM predicted the previously known area of distribution for *E. hirundinacea* including areas of Mexico’s Atlantic Slope, the Isthmus of Tehuantepec, Yucatan Peninsula, Guatemala, El Salvador, Belize, Honduras, Nicaragua, and Costa Rica (Fig. 5). The ENM predicted a suitability reduction throughout the area of the Nicaragua Depression. The paleodistributions indicate reductions in the geographic distribution area of *E. hirundinacea*. The Last Interglacial ENM projection predicted suitability in areas of the Atlantic Slope of Mexico and in Central America, in northeastern Nicaragua. The Last Glacial Maximum projections predicted small patches in Panama, Costa Rica, and Belize and larger patches of suitability on the Atlantic Slope of Mexico. For the Holocene, the suitability areas extended, however, still they are patches in Mexico and Central America (from Guatemala to Costa Rica). The demographic analysis using the Extended Bayesian Skyline Plots had an effective sample >200, for the demographic information this analysis showed that the population of *Euphonia hirundinacea* tended to increase since 50,000 years ago, with bigger growth in the past 20,000 years (Fig. 6). The ABBA-BABA test was significant for hybridization between *E. h. hirundinacea* and *E. h. gnatho* (see Table 3), with a significant Peterson D.

The G-PhoCS Analysis indicated that the two populations diverged 2,668,739 (95% HPD: 121,739–4,026,086) years ago. It also indicated that both populations have low migration rates (see Table 4), contrasting with the ABBA BABA results. Also, this demographic analysis suggests a large ancestral population with ∼22,304,347.83, and asymmetrical effective population size.

### Genetic distances and its relationship with geographic, environmental and phenotypic distances

The results of the MRM analysis showed that genetic distance had a significant positive relationship with the geographic distance (*R*^2^ = 0.05 *P* = 0.008, *F* = 39.13 *P* = 0.008) (Table 5). There was no significant relationship between genetic distance, temperature and precipitation variables (See Table 5). For females there was a statistically significant positive relationship between genetic and phenotypic distance (*R*^2^ = 0.08 *P* = 0.027, *F* = 32.91 *P* = 0.027), specifically for the morphometric variables (*R*^2^ = 4.09E-03 *P* = 0.005). For males, there was no significant relationship between phenotype differences and genetic distance (*R*^2^ = 0.03 *P* = 0.31 *F* = 13.15 *P* = 0.31). Finally, in the multiple regression model –which incorporated geographic, phenotypic, and environmental variables –morphometric distances remained statistically significant, showing the same patterns as before.

### Phenotypic distances and their relationship with geographic and environmental distances

The results of MRM analysis for color PC1 and wing chord did not show a significant relationship with the geographic distance and environmental variables (Tables S7 and S8). The MRM analysis for individual color variables only showed a positive relationship with geographic distance for the variable H3 (Hue) in the throat patch for females (*R*^2^ = 0.15 *P* = 0.001* *F* = 31.77 *P* = 0.001*) (Table S8), however, the R^2^ value is small, so this result has to be taken with cautions.

## Discussion

This study investigates the population genetic structure, demographic history, and distribution patterns of *Euphonia hirundinacea*, as well as the associations between phenotypic traits and geographic and ecological variables. Our results reveal genetic differentiation between two populations, shaped primarily by isolation by distance, and corresponding to the currently recognized subspecies *E. h. hirundinacea* and *E. h. gnatho*. The geographic break underlying this genetic divergence aligns with the Nicaragua Depression, which may have acted as a semi-permeable barrier. Pleistocene climatic reconstructions suggest the possibility of secondary contact, supported by demographic analyses indicating some degree of gene flow. Certain morphological traits, such as tail length, differ significantly between the two genetic groups. Correlation analyses indicate that geographic distance partially explains the observed genetic structure. Notably, some female specific traits (*e.g.*, wing chord) are associated with both genetic and geographic distance.

**Table 3:** ABBA-BABA tests for *E*. *hirundinacea*.

P1	Sister group (*E. laniirostris*)
P2	*E*. *h*. *hirundinacea*
P3	*E*. *h*. *gnatho*
Patterson’s D statistic	0.9569
Z-score	322.108
*P*-value	<0.05
D jackknife mean	0.9569
Admixture proportion	0.9512 (95%= 0.9424–0.9601)

**Table 4:** Population divergence times estimated by the G-PhoCS analysis.

Ancestral Ne *E. h. hirundinacea*/*gnatho*	≈ 22,304,347.83 (95% HPD: 21,038,043.48–23,809,782.61)
Ne *E. hirundinacea*	≈ 18,945,652.17 (95% HPD: 2,907,608.70–27,445,652.17)
Ne *E. gnatho*	≈ 3,592,391.30 (95% HPD: 188,043.48 5,260,869.57)
Divergence time *E. h. hirundinacea*/*gnatho*	∼2,668,739.13 (95% HPD: 121,739.13–4,026,086.96)
Migration rate by generation *E. h. hirundinacea* ->*E. h. gnatho*	0.000044251284
Migration rate by generation *E. h. gnatho* ->*E. h. hirundinacea*	0.00000845071975
Data-ld-ln	−120.9821
Full-ld-ln	−545 232.932

**Table 5:** MRM analysis for genetic, geographic, environment and phenotype distances for *Euphonia hirundinacea* data.

Variable	Coefficient	*P*
*Genetic ∼geographic distance R2 = 0.05P* = 0.008*F = 39.13*P* = 0*.008*		
Geographic distance	1.49E−08	0.008*
*Genetic ∼phenotype: color + morphometric*		
*Females R2 = 0.08P* = 0.027*F = 32.91*P* = 0.027*		
Morphometric (wing chord)	4.09E−03	0.005*
Color (PC1)	1.71E−03	0.37
*Males R2 = 0.03P* = 0.31*F* = 13*.15P* = 0*.31*		
Morphometric (wing chord)	2.17E−03	0.21
Color (PC1)	1.49E−03	0.44
*Genetic ∼geographic distance + morphometric + color + temperature + precipitation*		
*Females R2 = 0.13P* = 0.048*F = 23.60*P* = 0.01*		
Geographic distance	1.42E−08	0.006*
Morphometric (wing chord)	3.73E−03	0.005*
Color (PC1)	2.23E−03	0.20
Temperature	1.27E−03	0.40
Precipitation	−3.07E−06	0.57
*Males R2 = 0.10P* = 0.10*F* = 16*.82P* = 0*.10*		
Geographic distance	1.50E−08	0.008*
Morphometric (wing chord)	2.12E−03	0.199
Color (PC1)	2.07E−03	0.26
Temperature	1.62E−03	0.293
Precipitation	−4.04E−06	0.482

**Notes.**

The asterisks indicate statistically significant results with a *p*-value < 0.05.

### Genetic structure and isolation by distance

Our analyses reveal moderate genetic structure in *Euphonia hirundinacea*, shaped by isolation by distance (IBD) and geographic barriers. The PCA, explaining 17.1% of cumulative genetic variance (PC1-PC3), distinguished populations north and south of the Nicaragua Depression, suggesting localized differentiation. This was corroborated by our phylogenetic hypothesis, which recovered two well-supported divergent clades corresponding to Tamaulipas–northern Nicaragua (*E. h. hirundinacea*) and the Nicaragua Depression–Costa Rica (*E. h. gnatho*). The genetic assignment results from STRUCTURE support the presence of two genetic groups and evidence of admixture. While Principal Component Analysis (PCA) suggested a visual separation of the Nicaragua Depression population, the STRUCTURE analysis did not recognize the Nicaraguan samples as a separate population. Instead, this analysis assigned them to a single cluster along with samples spanning from Southeast Mexico to Nicaragua. This result suggests that the Nicaraguan Depression population shares a significant proportion of ancestry with northern samples, indicating that the population structure suggested by the PCA does not represent the entirety of the genetic variation. Additionally, the STRUCTURE graphic reveals that samples originating from the extremes of the geographical distribution of the *E. hirundinacea* are the most differentiated. The significant IBD signal (MMR results: *R*^2^ = 0.05, *p* = 0.008) supports that part of the genetic variance can be explained with the increasing of geographic distance. The STRUCTURE plot (Fig. 3) supports this, revealing that while northern and southern individuals did not share ancestry, those from central regions show clear evidence of admixture.

Notably, some morphological traits align with genetic divergence between *E*. *h*. *hirundinacea* and *E*. *h*. *gnatho*. However, the multivariate analysis suggests that in males only a small part of the variation can be explained by this groups. This pattern has been found in other Mesoamerican birds where phenotypic variation differs from the phylogeographic structure (*e.g.*, García et al., 2016; Miller et al., 2011; Rocha-Moreira, Hernández-Baños & Smith, 2020; Sánchez-González et al., 2021).

### The Nicaragua depression: a semi-permeable barrier for *Euphonia hirundinacea*

In contrast with several studies that identify the Nicaragua Depression as a strict biogeographic barrier (*e.g.*, Castillo-Chora et al., 2021; d’Horta et al., 2013; Puebla-Olivares et al., 2008; Rocha-Méndez et al., 2018), our ABBA-BABA and STRUCTURE analyses detected gene flow across this region. This permeability could reflect two phenomena. The first is Pleistocene dynamics: with fragmented habitats during the glacial epochs, followed with secondary contact during interglacial (∼130 kya), a recurrent pattern in Mesoamerica lowland taxa (McLaughlin & Miller, 2022; Miller et al., 2011; Sánchez-González et al., 2021). The second is historical connectivity: the species’ large effective population size (demographic analyses) and vagility have facilitated intermittent gene flow despite reduced suitability in the Depression during the LGM and Holocene (according to the ENMs), as has occurred in other birds (*e.g.*, *Saucerottia* complex species Jiménez & Ornelas, 2016).

### Population divergence, demographic history and paleodistribution modeling

The Pleistocene origin of divergence ∼2,668,739.13 (95% HPD: 121,739.13 –4,026,086.96) suggested a first separation between the groups of *E*. *h*. *hirundinacea* and *E*. *h*. *gnatho*, this result suggest a first separation at the beginning of the Pleistocene. According with biogeographic studies the linage of *E*. *hirundinacea* arrived to Mesoamerica from Panama Isthmus (Vázquez-López et al., 2024). Likely, the isolation occurred during the dispersion to northern areas of North America. The ENM results indicate that during the Pleistocene climatic change the habitat suitability had been reduced during the LIG and LGM, these results suggest that more than one isolation event can occurred with the populations of *E*. *hirundinacea*.

Our demographic analysis suggests a subsequent population expansion (EBSP: ∼20 kya to present) and secondary contact (ABBA-BABA) likely resulted from Holocene habitat reconnection, as ENMs predict increasing suitability. This fragmentation-expansion pattern is consistent with other Mesoamerican avian phylogeographic patterns (Espinosa-Chávez et al., 2024; Miller et al., 2011).

*Euphonia hirundinacea* occupies a wide variety of Mesoamerican lowland habitats—from intact forest to human-disturbed areas, including second-growth forest, forest edge, and isolated trees in pastures—is characterized by high adaptability (Howell & Webb, 1995; Whitman, 2020). This tolerance to habitat alterations may have further buffered it against prolonged isolation, enabling rapid recolonization during favorable periods. Although mostly resident, some regional movements suggest this species may be migratory or a non-breeding visitor (Binford, 1989). Therefore, its high vagility could be related to the admixture signal detected in the structure and demographic analysis, and it may also play an important role in recolonizing areas. These characteristics of *Euphonia hirundinacea* could explain the population pattern of two populations with admixture, and that isolation by distance partially explains the population structure.

The G-PhoCS analysis performed well according to ESS values (>200). However, we still recommend that the large interval of divergence times between the two populations (∼2,668,739.13, 95% HPD: 121,739.13–4,026,086.96) be interpreted with caution, as this wide interval could be a consequence of the gene flow after divergence that was detected by other methods. Although G-PhoCS did not detect continued migration, the ABBA-BABA test and structure analysis detected secondary contact between the two populations, and the IBD pattern can affect the performance of coalescent analysis.

### Relationships among genetic, geographic, environmental, and phenotypic distances

The MRM analysis revealed a significant relationship between wing chord measurements and genetic distances in females, suggesting potential sex-specific selection pressures or genotype-phenotype correlations. Notably, we found a significant association between female throat coloration (Hue, H3) and geographic distance; 15 percent of the variation in this trait is explained by geographic distance (*R*^2^ = 0.15, *p* = 0.001) (Table S8). This result is in line with the previously documented clinal variation in this trait across the species’ range (Phillips, 1966). Specifically, females exhibit a gradual intensification of yellow throat coloration from Chiapas to Costa Rica, a pattern that is consistent with isolation by distance. The clinal variation in female throat coloration (*e.g.*, Chiapas to Costa Rica) parallels patterns observed in other Neotropical birds, where chromatic divergence is decoupled from environmental gradients and associated with geographic distance (IBD) (*e.g.*, *Habia rubica* Ramírez-Barrera et al., 2019). Interestingly, the positive correlations between genetic distance and geographic distance for two female-specific traits imply that female-mediated processes may be associated with population differentiation along the geographic distribution in this species. However, neither geographic nor environmental distances showed significant relationships with other color variables (including color PC1), suggesting that alternative mechanisms beyond isolation by distance or environment are likely involved in these morphological variations. This is particularly interesting in conjunction with the lack of association between male phenotype variation (morphometric or color) and geographic, genetic, or environmental distances. This pattern differs from those observed in other Neotropical birds, where male phenotypes often are consistent with strong sexual selection combined with isolation by distance (*e.g.*, *Manacus,* Stein & Uy, 2006), female mate choice across spatial scales (*e.g.*, *Chiroxiphia*, Durães, Loiselle & Blake, 2009), or local adaptation and neutral drift (De Abreu, Schietti & Anciães, 2018). Furthermore, this lack of spatial or genetic association in males is associated with the dispersion of individuals between populations across the distribution range, as is suggested by the structure and admixture results. Lastly, despite some limitations, such as the sample size for the *E*. *h*. *gnatho* group, our study provides a valuable framework for exploring phenotypic variation. While fine-scale ecological factors may not be fully captured, our results establish an important work for future, more detailed investigations.

## Conclusions

This study reveals that *Euphonia hirundinacea* comprises two genetic groups corresponding to the recognized subspecies *E. h. hirundinacea* and *E. h. gnatho*. Overall, the genetic differentiation across the species range is partially explained by isolation by distance (IBD), with the Nicaragua Depression acting as a semi-permeable biogeographic barrier shaped by Pleistocene climatic fluctuations, allowing gene flow between populations. Phylogenomic and demographic analyses suggest an early Pleistocene divergence (∼2,668,739 years ago) with subsequent population expansion and secondary contact during Holocene (∼20,000 years ago). Specific female traits, such as wing chord and throat coloration, show significant correlations with genetic and geographic distances. In contrast, male phenotypic traits exhibit no clear association with spatial or genetic factors, which may be attributed to higher male dispersal. Our findings highlight the importance of geographic and demographic processes in shaping population structure and divergence within *E. hirundinacea*.

##  Supplemental Information

10.7717/peerj.20916/supp-1 Supplemental Information 1 Morphometric, color and sexual data Supplemental informations, including GBIF points, morphometric, color and sexual data

## Additional Information and Declarations

### Field Study Permissions

The following information was supplied relating to field study approvals (*i.e.*, approving body and any reference numbers): The field collection permit was provided by Instituto Nacional de Ecologia, SEMARNAT, Mexico (FAUT-0169).

### Data Availability

The following information was supplied regarding data availability: The raw data are available in GenBank: PRJNA1292171. *Euphonia violacea* (Accession number SAMN30618678) and *Euphonia laniirostris* (Accession number: 390050) and *Euphonia minuta* (Accession number: SAMN30630219).
https://www.ncbi.nlm.nih.gov/biosample/30618674
The raw data of color and morphometric measures and alignment is available at figshare: Vázquez, Melisa (2025). Raw DNA (sra), color and morphometric data of *Euphonia hirundinacea* (Aves: Fringilidae). figshare. Dataset. https://doi.org/10.6084/m9.figshare.29815412.v3.
